# Sensitivity and specificity of parallel or serial serological testing for detection of canine *Leishmania* infection

**DOI:** 10.1590/0074-02760150364

**Published:** 2016-03

**Authors:** Mauro Maciel de Arruda, Fabiano Borges Figueiredo, Andreza Pain Marcelino, José Ronaldo Barbosa, Guilherme Loureiro Werneck, Elza Ferreira Noronha, Gustavo Adolfo Sierra Romero

**Affiliations:** 1 Universidade de Brasília, Núcleo de Medicina Tropical, Brasília, DF, Brasil; 2 Fundação Oswaldo Cruz, Instituto de Pesquisa Clínica Evandro Chagas, Laboratório de Pesquisa Clínica em Dermatozoonoses em Animais Domésticos, Rio de Janeiro, RJ, Brasil; 3 Fundação Ezequiel Dias, Belo Horizonte, MG, Brasil; 4 Universidade do Estado do Rio de Janeiro, Instituto de Medicina Social, Rio de Janeiro, RJ, Brasil

**Keywords:** dogs, sensitivity, specificity, visceral leishmaniasis, Leishmania infantum, serology

## Abstract

In Brazil, human and canine visceral leishmaniasis (CVL) caused by*Leishmania infantum* has undergone urbanisation since 1980, constituting a public health problem, and serological tests are tools of choice for identifying infected dogs. Until recently, the Brazilian zoonoses control program recommended enzyme-linked immunosorbent assays (ELISA) and indirect immunofluorescence assays (IFA) as the screening and confirmatory methods, respectively, for the detection of canine infection. The purpose of this study was to estimate the accuracy of ELISA and IFA in parallel or serial combinations. The reference standard comprised the results of direct visualisation of parasites in histological sections, immunohistochemical test, or isolation of the parasite in culture. Samples from 98 cases and 1,327 noncases were included. Individually, both tests presented sensitivity of 91.8% and 90.8%, and specificity of 83.4 and 53.4%, for the ELISA and IFA, respectively. When tests were used in parallel combination, sensitivity attained 99.2%, while specificity dropped to 44.8%. When used in serial combination (ELISA followed by IFA), decreased sensitivity (83.3%) and increased specificity (92.5%) were observed. Serial testing approach improved specificity with moderate loss in sensitivity. This strategy could partially fulfill the needs of public health and dog owners for a more accurate diagnosis of CVL.

Visceral leishmaniasis (VL) in the Americas is a serious parasitic disease caused by the protozoan *Leishmania infantum* (Romero & Boelaert 2010, Ferreira et al. 2012). The World Health Organization (WHO) includes the leishmaniases among the most neglected tropical diseases, defined as expanding infections, for which no adequate control instruments are available (WHO 2010a). The transmission of this disease in the Americas occurs mainly through the bite of female sandflies of the species*Lutzomyia longipalpis* (Lainson & Rangel 2005), *Lutzomyia cruzi* (Missawa et al. 2011), and *Lutzomyia evansi* (Travi et al. 1990).

In Brazil, human and canine VL (CVL) are endemic and has undergone a continuous process of urbanisation since 1980, constituting a public health problem because of their wide distribution in the country and dispersal to regions considered unaffected as well as the severity of their clinical forms, which can cause death in the absence of proper and timely treatment (Marzochi et al. 2009, Carranza-Tamayo et al. 2010, Tonini et al. 2012).

The close relationship between humans and domestic dogs, the sharp cutaneous parasitism in dogs, the proven ability of infected dogs to infect sandflies, and the occurrence of canine enzooty preceding human cases and consistent correlation between canine seroprevalence and the risk of human disease development suggest that domestic dogs are the main reservoir of *L. infantum* in urban areas (Belo et al. 2013)*.* In this sense, the WHO recommends, prior to the onset of control activities, that special attention should paid to the study of frequency and distribution of *L. infantum* in dogs by means of serological surveys (WHO 2010b).

The Program for Surveillance and Control of Visceral Leishmaniasis in Brazil aims to reduce the number of human cases and deaths through the early diagnosis and treatment of human cases and the control of vectors and urban reservoirs. In Brazil, one of the control measures focused on urban reservoirs is the euthanasia of seropositive dogs, with the purpose of reducing transmission to humans. The WHO considers serological screening and euthanasia of seropositive dogs among human VL control measures; however, they indicate flaws in the effectiveness of this action, in part because of the lower accuracy of the diagnostic tests for CVL (WHO 2010b).

The euthanasia of infected dogs has been the subject of much controversy; however, there is consensus regarding the need for improved serological tests that can more accurately estimate the magnitude of infection in the canine population and its evolution over time, particularly when this population is targeted for transmission control interventions (Romero & Boelaert 2010, Costa 2011).

The Brazilian zoonosis control program recommended until 2012 the enzyme-linked immunosorbent assay (ELISA) and indirect immunofluorescence assays (IFA) as the screening and confirmatory methods, respectively, for CVL diagnosis. Both tests use crude antigen of *L. major* produced by the Bio-Manguinhos Laboratory at Oswaldo Cruz Foundation (Fiocruz), Rio de Janeiro, Brazil (de Arruda et al. 2013). Nevertheless, the strategy of combined serological tests for the diagnosis of CVL was not subjected to a comprehensive validation study using a large sample of dogs from endemic areas.

More recently, a dual-path platform (DPP) test has been evaluated (Schubach et al. 2014). DPP was developed as a promising test devoted to rapid diagnosis under field conditions. However, DPP lower sensitivity could hamper its use in this scenario and at least one study suggest its inclusion in serial testing combined with an ELISA test (Coura-Vital et al. 2014).

Parallel or serial testing has been recognised as a strategy to improve the diagnostic accuracy of tests used for screening purposes (Gordis 2000). Then, the objective of this study is to estimate the accuracy of ELISA and IFA tests (Bio-Manguinhos) applied in serial or parallel combination and analyse the positive predictive values (PPV) of these approaches in variable prevalence levels of*Leishmania* infection in the Brazilian scenario.

## MATERIALS AND METHODS

**Fig. 1:**
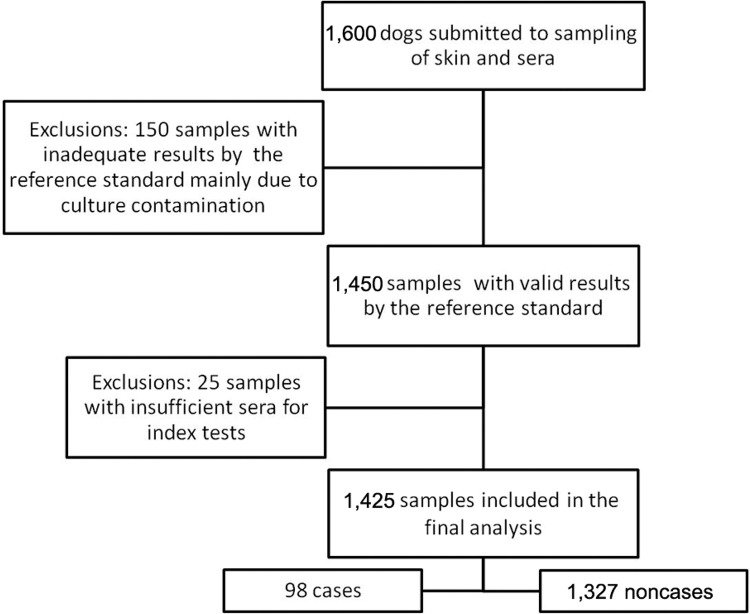
: flow diagram of the inclusion procedures. Algorithm for inclusion and exclusion of canine serum samples for the validation of enzyme-linked immunosorbent assay and indirect immunofluorescence assay with *Leishmania major* antigens.

*Study population* - A panel composed of 1,425 sera collected between 2008-2010, as previously reported by de Arruda et al. (2013), was used for the present study. Briefly, animals were consecutively recruited in four Brazilian cities without prior clinical assessment or laboratory diagnosis by any of the reference standard or index tests. Exclusion criteria were pregnancy, aggressive dogs precluding the diagnostic procedures, and owners refusing the informed consent. One hundred seventy-five samples were considered inappropriate for the study purpose: 150 were excluded due to inconclusive results in parasitological tests, mainly contamination of skin cultures, and 25 due to the small volume of serum available for the index tests procedures (Fig. 1).

*Reference standard* - The reference standard was applied to all dogs included in the study. Reference standard comprised the results of parasitological examination of skin samples by the direct visualisation of parasites in histological sections stained with haematoxylin-eosin (H&E), immunohistochemical test, or isolation of the parasite in culture medium. This approach was used to improve the sensitivity of the reference test. Skin lesions (when present) or healthy skin were collected from all for the selected animals. The skin samples were submitted to parasitological examination by the direct visualisation of parasites in histological sections stained with H&E, immunohistochemical test, and isolation of the parasite in culture medium (Madeira et al. 2009, Quintella et al. 2009, d, de Almeida et al. 2011). Animals which tested positive in at least one of the tests above were considered cases. Animals which tested negative in all the parasitological tests were considered no-cases (controls). The samples were processed at the Laboratory of Leishmaniasis Surveillance, Evandro Chagas Research Institute (Fiocruz), a national referral centre for the parasitological diagnosis of leishmaniasis. Technicians conducting the reference standard tests were blind to the results of the index tests.

*Index tests* - The protocols for ELISA (*Leishmania major*) and IFA (*L. major*) followed the manufacturer’s recommendations (Bio-Manguinhos, Brazil). The ELISA readings were performed with microplate spectrophotometer equipped with a 450 nm filter. ELISA cut-off point was twice the mean optical density (OD) of the negative controls present on the plate, as recommended by the manufacturer. Samples that showed OD values between the cut-off and 1.2 times the cut-off value were considered indeterminate and were retested. Samples that maintained the indeterminate status were considered negative, since euthanasia is not recommended for animals in these conditions. IFA cut-off point was ≥ 1:40, as recommended by the manufacturer, and reading was performed with an immunofluorescence microscope independently by two observers. For IFA we used the criterion of concordant results by two-observers as definite result, otherwise the test was considered negative. Serological tests were carried out at the National Reference Laboratory for Leishmaniasis, Ezequiel Dias Foundation, Belo Horizonte, state of Minas Gerais, Brazil, in March and April 2010. Technicians conducting serological tests were blind to the results of the reference standard.

*Data analysis* - The results obtained in the laboratory tests were organised in MS Excel spreadsheets and analysed using the software package SPSS 16 for Windows. Sensitivity, specificity, PPV, and negative predictive value were estimated individually and in combination, with their respective 95% confidence intervals (Gardner & Altman 1989). Sensitivity was calculated as the proportion of positive results obtained with the index test among cases. Specificity was calculated as the proportion of negative results obtained with the index among noncases. PPV was calculated as the proportion of true-positive results among all positive results obtained with the index test. NPV was calculated as the proportion of true-negative results among all negative results obtained with the index test.

*Ethics* - The project was approved by the Ethical Committee on Animal Use of Fiocruz under license L-38/08. All the owners signed the informed consent previous to the collection of the samples and skin sampling procedures were performed under sedation in accordance with the Brazilian rules for conducting research in animals. No adverse events were registered during the sample collection and processing in the lab.

## RESULTS

Reference standard tests were performed between 2008-2010. Index tests were performed in 2010. According to the reference standard, 98 (6.9%) samples were classified as positive (cases) and 1,327 (93.1%) as negative (noncases), for *L. infantum* infection.

Sixty samples tested indeterminate in the ELISA and were retested. After retesting, 27 samples tested positive and were definitively classified as being positive. Fourteen samples tested negative and 19 samples remained with indeterminate results, then 33 samples were definitively classified as being negative.

Similar sensitivity was obtained for ELISA (91.8%) and IFA (90.8%), and higher specificity was observed for ELISA (83.4%) compared to IFA (53.4%). When tests were combined in parallel, global sensitivity attained 99.2%, while global specificity dropped to 44.8%. When used in serial combination (ELISA followed by IFA), decreased sensitivity (83.3%) and increased specificity (92.5%) were observed (Table).

**TABLE:** Accuracy of enzyme-linked immunosorbent assay (ELISA) and indirect immunofluorescence assay (IFA), individually and combined in sequence or parallel, in serum samples of dogs from areas endemic for visceral leishmaniasis in Brazil, 2011

Accuracy	ELISA^*a*^ % (95% CI)	IFA % (95% CI)	Serial testing^*b*^ % (95% CI)	Parallel testing % (95% CI)
Sensitivity	91.8	90.8	83.3	99.2
	(86.3-97.3)	(84.2-97.5)	(75.6-91.0)	(75.6-91.0)
Specificity	83.8	53.4	92.5	44.8
	(81.8-85.7)	(51.6-55.1)	(87.1-97.9)	(87.1-97.9)
PPV	29.6	12.6	44.9	11.7
	(24.5-34.7)	(7.1-18.1)	(34.4-55.1)	(5.0-18.1)
NPV	99.3	98.7	98.7	99.8
	(98.8-99.8)	(98.2-99.3)	(96.4-100)	(98.9-100)

*a*: data on this column were reported previously in de Arruda et al. (2013); *b*: ELISA followed by IFA; CI: confidence interval; NPV: negative predictive value; PPV: positive predictive value.

**Fig. 2:**
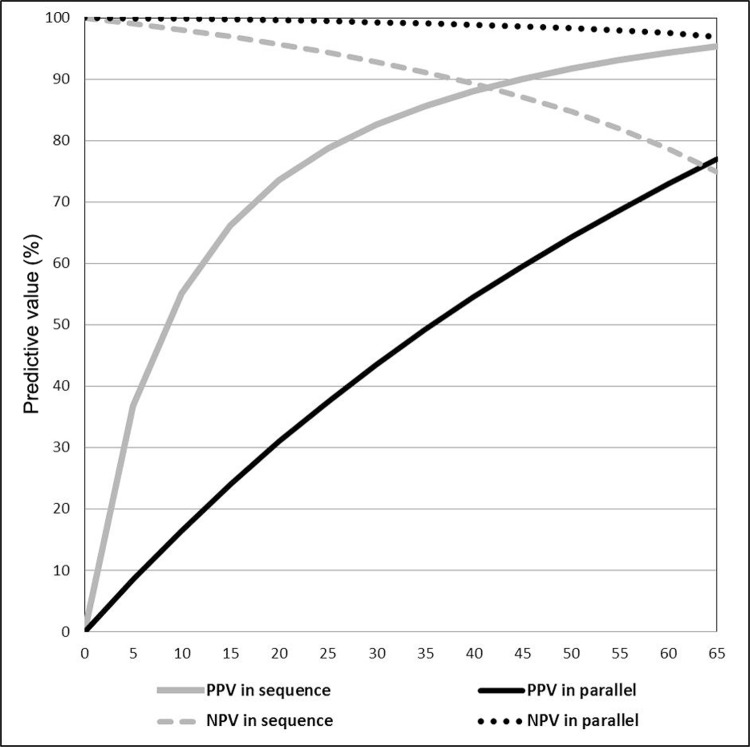
: sensitivity analysis of positive (PPV) and negative (NPV) predictive values of tests combinations for visceral leishmaniasis diagnosis for enzyme-linked immunosorbent assay (ELISA) and indirect immunofluorescence assay (IFA) (Bio-Manguinhos®) in serial (ELISA followed by IFA) or parallel combinations, according to prevalence levels of canine visceral leishmaniasis.

The analysis for scenarios with prevalence levels lower than 10% showed that the PPV becomes critical in these situations, predicting the classification of one or more noninfected dogs as seropositive for each infected dog correctly classified (Fig. 2).

## DISCUSSION

The prevalence of infected dogs living in endemic areas in Brazil shows wide variation: from 1-67% (Coutinho et al. 1985,Nunes et al. 1991, França-Silva et al. 2005, Monteiro et al. 2005, Mestre & Fontes 2007, de Arruda et al. 2013). Control of the canine reservoir by means of serological surveys for identification of infected dogs and their subsequent euthanasia has been developed within a scenario considered inappropriate, mainly because of the limitations of diagnostic methods (Costa et al. 2013). The assessment of canine seroprevalence in endemic areas can generate many doubts depending on the sensitivity and specificity of the tests used, which have shown variable accuracy between many validation studies (Porrozzi et al. 2007). These variations could be associated to the specific characteristics of the target population of dogs assessed and the sampling strategies used for the validation process. Other sources of variation originate from the technical characteristics of the test, the proficiency of the laboratory, the reference standard selected for comparison, and the cut-off point used for the interpretation of results. Biological factors may also affect the accuracy of serological tests, and sensitivity can vary according to the stage of infection or the immune status of the host, while reduction in specificity can be explained by the cross-reactivity with other infectious agents, or when this property is estimated in dogs that are actually infected but are not adequately detected by the reference standard (Greiner & Gardner 2000).

The use of highly sensitive tests, which detect the largest possible number of infected animals, would be the most recommended for public health actions that aim to reduce transmission. Moreover, highly specific tests are desirable for animal protection associations, veterinary practitioners, and dog owners who seek the safety that only truly infected animals be euthanised. Diagnostic tests used in combination can improve diagnostic accuracy and could meet the demands of public health interventions and dog owners living in endemic areas.

Our results showed lower specificity of IFA compared to ELISA. Although the idea of higher specificity of IFA compared to ELISA is a common expectation, it is by no means an obligate premise. Most of this perception has been based on results from studies conducted with pre-screened samples that are clearly biased for improving specificity. In the present case, the specificity of both tests was not as good as could be expected and we could explain this finding as a possible effect of our study design without any screening, clinical or serological, before sample collection. Also, we recognise that the imperfection of the reference standard could have had a selective deleterious effect on IFA specificity, but unfortunately we do not have any scientific data to prove that hypothesis. We did not perform any comparison of test performance between samples from different regions but all the sampling procedures were homogeneous and tests were conducted in just one reference laboratory.

The results presented herein are consistent with those obtained by Lira et al. (2006) and reported a significant improvement in test sensitivity from 72% (ELISA) and 68% (IFA) to 92%, with a decrease in specificity from 87.5-75% when used in parallel combination. In the same study there was a gain in specificity (100%) and a drop to 48% in sensitivity when tests were used in sequence. These authors recommend the use of tests in parallel combination for high-prevalence areas, reducing the number of infected animals that could remain in the environment; on the other hand, for areas of low prevalence, the use of tests in serial combination was recommended as most appropriate.

Our sensitivity analysis, simulated for different prevalence levels, reinforce the sense of these authors’ recommendations. Taking into account the actual prevalence of infection in the sera panel used in the present study, the strategy of applying the tests in serial combination would be the most appropriate to similar Brazilian scenarios. The combined use, in spite of losing sensitivity, allows the improvement of the PPV, lowering the unwanted consequences of euthanasia of dogs that are actually not infected. Both approaches, serial and parallel, showed high NPV from 0-40% prevalence rates. NPV drops below 90% with the serial approach in scenarios with prevalence higher than 40%. Then, as expected, the parallel approach is a very reasonable strategy for identifying noninfected dogs no matter the prevalence of infection in the population were, and the serial approach could be reasonable for that purpose for scenarios with prevalence lower than 40%.

The recent mathematical modelling developed by Costa et al. (2013) reveals that the sensitivity and specificity of the diagnostic tests used for the control of CVL would determine the success or failure of the strategy for elimination of seropositive dogs. In that model, in scenarios with low and moderate transmission, the elimination of dogs based on the result of a test with 90% sensitivity and specificity of 80%, applied to the clinically suspected and apparently healthy canine population, would have a significant impact on transmission in the long term. This study also suggests that, in order to keep a screening and culling strategy of only clinically suspected dogs with the same long-term impact, tests with sensitivity of at least 90% would be necessary, and that the isolated canine culling strategy would not be sufficient to cause an impact in scenarios with higher transmission (Costa et al. 2013). Our results demonstrated that it is possible to improve diagnostic accuracy through sequentially combined tests, achieving sensitivity and specificity that, according to the mathematical model previously described, would be appropriate for dog-culling intervention in scenarios with low-to-moderate transmission. However, when the PPV of the combination of tests in the scenario of actual prevalence found in the study is taken into account, one noninfected dog would be diagnosed and euthanised for each infected dog correctly diagnosed and euthanised. Therefore, the data herein presented are of utmost importance to support the predictions obtained by mathematical modelling studies with valid parameters of sensitivity and specificity of the diagnostic tests used.

One limitation of the present study was related to the imperfection of the reference standard which could be not sensitive enough with the consequent worsening of the specificity estimates for the studied tests. Also, the use of *L. major* antigen in the IFA was not extensively validated as a substitute of the homologous *L. infantum* antigen as it is the case of the ELISA assay. Another drawback of the serial testing approach based on the two tests studied is the operational problem of delaying to remove infected dogs, which only rapid tests applied directly in the field, would be able to solve. However, the accuracy demonstrated by rapid tests is still not high enough to be singly applied in the diagnosis of CVL (Grimaldi Jr et al. 2012, Schubach et al. 2014) and point-of-care accurate molecular tools are not currently available.

These results demonstrate that it is possible to improve the diagnostic accuracy through the use of a combined serological testing approach and that the accuracy of the two tests combined in sequence reached reasonable sensitivity and specificity for the control of CVL in scenarios of low or moderate transmission.
